# Attention-guided saptio-temporal feature fusion for robus video surveillance anomaly detection

**DOI:** 10.1038/s41598-026-36130-z

**Published:** 2026-02-10

**Authors:** S. Deepa Nivethika, Shreyash Joshi, Kshitij Verma, V. Aishwarya, Vimal Varshan Srinivasan, M. Senthil Pandian, Prabhakaran Paulraj

**Affiliations:** 1 School of Computer Science and Engineering, Vellore Institute of Technology, Chennai, India; 2 School of Mechanical Engineering, Vellore Institute of Technology, Chennai, India; 3 School of Civil Engineering, Vellore Institute of Technology, Chennai, India; 4 Department of ECE, St. Joseph University in Tanzania, Dar es Salaam, Tanzania

**Keywords:** Video surveillance, Anomaly detection, Spatio-temporal feature fusion, Attention mechanism, ConvLSTM, Temporal consistency modeling, Theft detection, UCF-crime dataset, ShanghaiTech dataset, YOLO-v4, Engineering, Mathematics and computing

## Abstract

Dynamic object detection and tracking are essential components of intelligent video surveillance systems, enabling real-time monitoring and early identification of anomalous activities. Existing approaches often rely on either spatial appearance modeling or temporal sequence analysis, which limits robustness in crowded and dynamically evolving scenes. This study first evaluates representative spatial and temporal baseline models for theft detection, including an EfficientNetV2B0–HOG framework and a ConvLSTM-based temporal model, which achieve F1-scores of 0.86 and high recall but suffer from limited temporal consistency and sensitivity to data imbalance. To address these limitations, we propose an attention-guided spatio-temporal hybrid framework, referred to as HybridModel-1, which integrates object-level spatial detection with temporal motion modeling. The proposed model incorporates an Adaptive Feature Fusion Module (AFFM) to dynamically emphasize salient spatial features and a Temporal Confidence Reweighting Loss to suppress temporally inconsistent predictions. Evaluated on large-scale surveillance benchmarks including UCF-Crime, ShanghaiTech, and DCSASS, the proposed framework achieves an accuracy of 87.6%, a precision of 95.6%, a recall of 77.1%, and a ROC–AUC of 0.96, outperforming standalone spatial and temporal baselines. Ablation studies further confirm the effectiveness of the proposed fusion and temporal consistency mechanisms, demonstrating the model’s suitability for real-time surveillance applications.

## Introduction

In recent years, dynamic object detection and tracking have become integral components of modern surveillance systems, driven by the growing demand for public safety and security in urban and commercial environments. Traditional surveillance infrastructures rely heavily on continuous human monitoring of video feeds, which is often inefficient and susceptible to fatigue, delayed reactions, and missed events. As surveillance networks expand in scale and complexity, automated video analysis systems have become essential for identifying suspicious activities and enabling timely responses to potential security threats^1,2^.

Among various surveillance applications, theft detection has emerged as a particularly critical task due to its prevalence in both public spaces and retail environments^3,4^. Detecting such incidents in real time requires systems that can not only localize objects and individuals accurately but also understand motion patterns and behavioral context within dynamic scenes^5,6^.

Recent advances in machine learning and deep learning have significantly improved the capabilities of automated surveillance systems. State-of-the-art methods leverage convolutional neural networks and recurrent architectures to analyze video streams, recognize human activities, and identify anomalous behavior indicative of criminal actions^7,8^. Hybrid models, which combine multiple learning paradigms, have gained increasing attention for their ability to balance detection accuracy and computational efficiency^9–11^. However, despite these advancements, many existing approaches struggle to generalize across diverse environments, handle occlusions effectively, and suppress false positives in crowded or visually complex scenes^12,13^.

Abnormal human activity detection plays a central role in intelligent surveillance systems by identifying deviations from expected or routine behavior that may signal security risks or emergency situations^14–16^. Such activities can broadly be categorized into short-term and long-term events. Short-term activities, such as sudden running, fighting, or object grabbing, involve rapid motion changes and are typically detected through immediate spatial and motion analysis^3^. In contrast, long-term activities such as loitering or unauthorized access require sustained observation and temporal reasoning to capture deviations from normal behavioral patterns over extended periods^17,18^. Effectively addressing both categories remains a challenging problem in real-world surveillance scenarios.

Motivated by these challenges, this work systematically explores and compares two established hybrid approaches for theft detection before introducing a more advanced detection framework. The first approach employs an EfficientNetV2B0 architecture combined with Histogram of Oriented Gradients (HOG) features to perform structured image-based theft detection^4,19^. While effective for static visual cues, this model exhibits limitations when handling occlusions and complex motion patterns. The second approach utilizes a Convolutional Long Short-Term Memory (ConvLSTM) network to process sequential video frames, enabling improved temporal awareness and motion-based reasoning^9^. Although this model enhances recall for abnormal events, it remains sensitive to data imbalance and temporal inconsistency.

Building upon the insights gained from these baseline models, we propose HybridModel-1, a spatio-temporal surveillance framework that integrates a ResNet-50 backbone with YOLO-v4 for object-level detection in untrimmed surveillance videos. Trained on the large-scale UCF-Crime dataset^15,20^, the proposed model aims to leverage strong spatial representations while maintaining temporal coherence across video sequences. By addressing both object detection and motion dynamics, HybridModel-1 is designed to improve robustness across multiple crime scenarios and dynamic environments, supporting real-time surveillance applications.

This paper conducts a thorough comparative analysis, elucidating the advantages and disadvantages of current hybrid designs, while illustrating how an improved spatio-temporal design can alleviate prevalent issues such as false positives, occlusion sensitivity, and temporal inconsistency. The results offer pragmatic insights for the design of more dependable surveillance systems and aid in the creation of flexible, precise, and scalable solutions for real-world security monitoring^1,7^.

Even with these improvements, there are still many outstanding problems that affect research in finding unusual behavior. It is still very important for real-world use^21^ to make sure that generalization works well in different lighting conditions, camera angles, and environmental circumstances. Data imbalance is another big problem because aberrant events are naturally rare relative to regular activity. This typically skews learning processes and makes detection less effective^3,9^. Also, in operational surveillance systems, it’s important to keep false positives to a minimum because too many false alarms might overburden security staff and make the system less effective overall^2,13^.

Looking ahead, future research directions focus on incorporating multi-modal information, such as audio signals or contextual metadata, to enrich anomaly detection capabilities^15^. Efforts are also being directed toward developing computationally efficient models optimized for real-time inference in large-scale surveillance networks^8,17^. Furthermore, self-supervised and weakly supervised learning strategies are gaining interest for their potential to improve generalization in scenarios where labeled data is limited^10^. Advancements in these areas are expected to further strengthen abnormal activity detection systems, enabling proactive and reliable security monitoring in complex environments.

While prior studies have combined ResNet backbones with YOLO detectors, these integrations are typically sequential and lack mechanisms to address feature redundancy and temporal instability. In contrast, the proposed HybridModel-1 introduces an Adaptive Feature Fusion Module (AFFM) that dynamically reweights spatial features and a Temporal Confidence Reweighting Loss (TCR-Loss) that enforces prediction consistency across frames. This dual-level innovation distinguishes the proposed framework from existing ResNet–YOLO pipelines by explicitly targeting the challenges of anomaly detection in untrimmed surveillance videos.

### Research gap and novelty

Although recent surveillance systems increasingly employ deep learning–based object detection and temporal modeling, existing approaches predominantly rely on either frame-level detectors or sequential models that treat spatial and temporal cues independently. Such pipelines often suffer from feature redundancy, temporally unstable predictions, and elevated false-positive rates in complex, untrimmed surveillance videos. Moreover, many hybrid frameworks combine multiple backbones without explicitly addressing how spatial semantics and detection-oriented features should be adaptively integrated or how temporal consistency can be enforced during training.

This work proposes a structured spatio-temporal surveillance framework to address existing gaps, introducing two key innovations: (i) an Adaptive Feature Fusion Module (AFFM) that dynamically reweights and integrates semantic and detection features to minimize background noise and highlight motion-relevant areas, and (ii) a Temporal Confidence Reweighting Loss (TCR-Loss) that ensures detection stability across consecutive frames by penalizing temporally inconsistent confidence variations. The suggested method is different from regular hybrid pipelines because it explicitly incorporates feature interaction and temporal coherence within a single detection framework. This makes it easier to find anomalies in real-world surveillance settings.

Even while object detection and video-based surveillance have come a long way, current approaches have evident problems when used on real-world, unedited surveillance films. Single-stage detectors like YOLOv4 and YOLOv8 work well in real time, but they mostly use spatial cues and don’t have ways to make sure that frames are consistent over time. On the other hand, transformer-based trackers and multi-stream designs, such as TransTrack and DETR versions, show better temporal reasoning but are much harder to compute, which makes them less useful in real-time surveillance settings. Additionally, current hybrid methods frequently integrate backbones and detectors sequentially, neglecting feature redundancy and temporal instability.

This work addresses these gaps by introducing an adaptive hybrid framework that explicitly enhances feature fusion and temporal consistency without sacrificing real-time performance. The proposed HybridModel-1 integrates an Adaptive Feature Fusion Module (AFFM) to mitigate redundancy between backbone and detector features, along with a Temporal Confidence Reweighting Loss (TCR-Loss) that penalizes temporally inconsistent predictions. Unlike prior hybrid models, the proposed design introduces architectural and loss-level innovations tailored for anomaly detection in surveillance videos, achieving improved robustness while maintaining computational feasibility.

## Literature review

Computer vision and deep learning algorithms for object detection, tracking, and recognizing unusual behavior have made automated surveillance systems much better. Early methods mostly used hand-crafted features and typical machine learning algorithms, which weren’t very scalable or robust when used in complicated real-world settings. With the advent of deep neural networks, modern surveillance systems have increasingly shifted toward learning-based methods capable of modeling spatial and temporal patterns directly from video data.

### Object detection-based surveillance approaches

Object detection plays a foundational role in video surveillance by enabling the localization of people and objects of interest within a scene. Single-stage detectors such as YOLO and SSD have gained popularity due to their real-time performance, making them suitable for large-scale surveillance applications. YOLO-v4, in particular, integrates a CSPDarknet backbone and feature pyramid structures to achieve high detection accuracy while maintaining low latency. Several studies have employed YOLO-based architectures for surveillance tasks such as theft detection, crowd monitoring, and anomaly localization, demonstrating strong spatial detection performance in controlled environments.

Recent works have further enhanced detection pipelines by incorporating stronger backbones or transformer-based architectures. For instance, YOLOv8 and DETR variants improve feature representation and detection precision through attention mechanisms and end-to-end training strategies. However, these approaches are often computationally expensive and may struggle with temporal reasoning when applied directly to long, untrimmed surveillance videos. Moreover, many detection-centric methods focus on frame-level predictions, leading to fragmented or temporally inconsistent alerts in continuous video streams.

### Temporal modeling for abnormal activity detection

To address the limitations of purely spatial detectors, temporal modeling techniques have been introduced to capture motion dynamics and behavioral patterns across video frames. Recurrent neural networks, particularly Long Short-Term Memory (LSTM) and Convolutional LSTM (ConvLSTM) architectures, have been widely used for video-based anomaly detection. ConvLSTM models preserve spatial structure while learning temporal dependencies, making them effective for recognizing motion-driven anomalies such as fighting, running, or theft-related actions.

Several studies have reported high recall rates using ConvLSTM-based frameworks on benchmark datasets such as UCF-Crime and ShanghaiTech. However, these methods often require fixed-length video sequences and extensive preprocessing, which complicates deployment in real-world surveillance systems where video durations vary significantly. Additionally, ConvLSTM models are sensitive to class imbalance, as abnormal events are relatively rare compared to normal activities, often leading to unstable predictions and increased false positives.

More recently, transformer-based temporal models and recurrent transformer hybrids have been proposed to improve long-range dependency modeling. While these methods show promising results, their computational complexity and memory requirements limit their applicability in real-time or edge-based surveillance scenarios.

### Hybrid and multi-stream surveillance models

Hybrid architectures that combine spatial object detection with temporal sequence modeling have gained increasing attention as a means to balance accuracy and efficiency. These approaches typically integrate convolutional backbones for feature extraction with temporal modules such as LSTMs, ConvLSTMs, or temporal convolutional networks. Multi-stream frameworks incorporating pose estimation, optical flow, or skeleton-based representations have also been explored to enhance activity recognition performance.

Recent studies have demonstrated that hybrid models outperform single-stream architectures by leveraging complementary spatial and temporal cues. For example, multi-stream systems combining RGB frames with motion or pose information have shown improved robustness in crowded and occlusion-heavy scenes. However, many existing hybrid frameworks rely on straightforward chaining of independent modules without adaptive feature fusion or temporal consistency enforcement. As a result, such systems may produce redundant features, suffer from false alarms, or exhibit unstable predictions across consecutive frames.

Additionally, several works employ heavy backbones or complex fusion strategies that increase inference latency, making them unsuitable for real-time surveillance deployment. Cross-dataset generalization remains another open challenge, as models trained on specific environments often fail to generalize effectively to unseen scenes with different lighting, camera viewpoints, or crowd densities.

### Identified research gaps

Despite the progress made in object detection, temporal modeling, and hybrid surveillance frameworks, several gaps remain in the current literature:

1. *Lack of adaptive feature fusion* Most hybrid models perform static or late-stage fusion of spatial and temporal features, without dynamically reweighting features based on scene context or motion relevance.
2. *Temporal inconsistency in predictions* Frame-level detection approaches often generate fluctuating outputs, leading to unstable alerts in continuous surveillance videos.
3. *Limited ablation and comparative analysis* Many studies report performance improvements without systematically analyzing the contribution of individual components or comparing against strong baselines.
4. *Practical deployment challenges* High computational cost, sensitivity to data imbalance, and poor generalization across datasets hinder real-world adoption.

### Positioning of the proposed work

Motivated by these limitations, the present work aims to advance hybrid surveillance modeling by introducing a spatio-temporal framework that explicitly addresses feature redundancy and temporal instability. Unlike prior approaches that rely on direct chaining of detection and temporal modules, the proposed method incorporates adaptive feature fusion and temporal confidence modeling to improve robustness in dynamic surveillance environments. By conducting extensive ablation studies and evaluating performance across multiple benchmark datasets, this work provides a clearer understanding of how hybrid architectures can be optimized for reliable, real-time abnormal activity detection.

## Datasets

To comprehensively evaluate the effectiveness and robustness of the proposed surveillance framework, experiments were conducted using multiple publicly available benchmark datasets widely adopted in abnormal activity and crime detection research. These datasets capture diverse surveillance scenarios, including variations in scene context, camera viewpoints, crowd density, and activity complexity.

For video-based experiments, each surveillance video was uniformly sampled into fixed-length frame sequences. A sequence length of T = 16 frames was used for ConvLSTM-based modeling, balancing temporal context and computational feasibility. Longer videos were segmented into overlapping sequences using a sliding window approach, while shorter clips were zero-padded to maintain consistency.

### DCSASS dataset

The DCSASS (Dataset for Crime Scene Analysis and Surveillance Systems) dataset is used to evaluate image-based theft detection under controlled conditions. It consists of labeled images representing normal and theft-related activities captured in indoor surveillance environments. The dataset provides clear visual cues and relatively structured scenes, making it suitable for assessing the performance of spatial feature extraction models.

In this work, the DCSASS dataset is used to train and evaluate the EfficientNetV2B0 combined with Histogram of Oriented Gradients (HOG) features. Images are resized to a fixed resolution and normalized prior to training. The dataset is split into training and testing sets following an 80:20 ratio to ensure fair evaluation. While effective for learning static appearance features, the dataset does not capture temporal motion patterns, highlighting the limitations of image-only approaches. Places365 dataset - https://www.kaggle.com/datasets/benjaminkz/places365.

### UCF-crime dataset

The UCF-Crime dataset is a large-scale video dataset designed for real-world crime detection in surveillance footage. It contains untrimmed videos spanning multiple crime categories, including theft, robbery, assault, and vandalism, as well as normal activities. Videos vary significantly in duration, lighting conditions, camera angles, and scene complexity, making the dataset highly challenging and representative of real-world surveillance scenarios.

In this study, the UCF-Crime dataset is employed to train and evaluate the proposed HybridModel-1. Videos are processed into frame sequences at a fixed frame rate, and object-level detections are extracted using YOLO-v4. To enable temporal modeling, fixed-length frame sequences are generated using a sliding window strategy, allowing the ConvLSTM-based temporal branch to handle variable-length videos effectively. The dataset is divided into training and testing subsets according to the standard split provided by the dataset creators. UCF Crime dataset - https://www.crcv.ucf.edu/projects/real-world/.

### ShanghaiTech campus dataset

The ShanghaiTech Campus dataset is a widely used benchmark for anomaly detection in crowded surveillance environments. It consists of videos captured from multiple cameras across different campus locations, featuring normal activities such as walking and gathering, as well as abnormal events including running, chasing, and fighting. The dataset emphasizes subtle motion variations and crowd dynamics, making it particularly suitable for evaluating temporal consistency and false positive suppression.

For experimental evaluation, videos from the ShanghaiTech dataset are processed into frame-level sequences and used primarily for cross-dataset validation. This enables assessment of the generalization capability of the proposed model when applied to environments and activity patterns not seen during training. Frame extraction and normalization procedures are kept consistent with those used for the UCF-Crime dataset to ensure comparability. ShanghaiTech dataset - https://svip-lab.github.io/dataset/campus_dataset.html.

### Dataset summary and usage

Table X summarizes the key characteristics of the datasets used in this study, including data modality, application focus, and evaluation purpose.

- *DCSASS* is used for image-based theft detection and baseline spatial analysis.
- *UCF-Crime* serves as the primary dataset for training and evaluating spatio-temporal crime detection models.
- *ShanghaiTech* is utilized to assess generalization performance and robustness in crowded surveillance scenes.

By evaluating the proposed framework across multiple datasets with varying characteristics, this study ensures a more comprehensive and reliable assessment of model performance under diverse real-world conditions.

## Methodology

This section presents the complete methodological framework adopted in this work for dynamic object detection, tracking, and anomaly analysis in surveillance videos. The methodology builds progressively from established hybrid models to the proposed HybridModel-1, ensuring continuity with prior approaches while introducing targeted innovations to address their limitations. Figure references throughout this section correspond to the existing workflow diagrams and architectural illustrations already presented.

### Overall system pipeline

The overall system follows a multi-stage pipeline, as illustrated in the existing methodology figures. Raw surveillance videos are first preprocessed and decomposed into frames. These frames are then analyzed using spatial feature extraction, temporal modeling, and object detection modules. Finally, frame-level predictions are aggregated over time to generate anomaly scores for surveillance decision-making.This structured pipeline enables the system to capture both instantaneous visual cues and long-term behavioral patterns, which are essential for reliable crime and theft detection in real-world environments.

### Video preprocessing and frame extraction

**Fig. 1:**
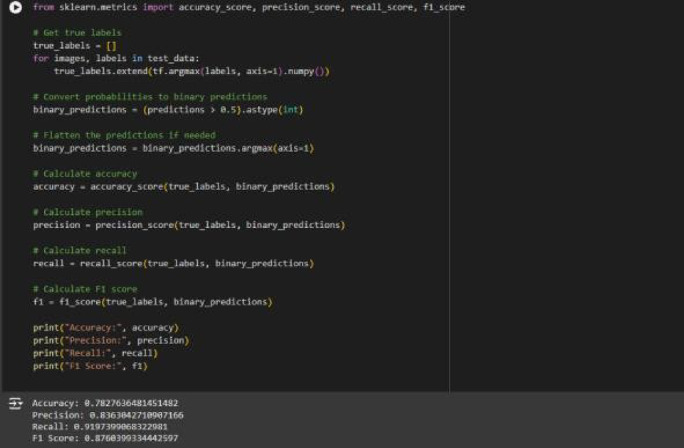
EfficientNetV2B0 with HOG analysis: python implementation for evaluating model performance, showcasing the computation of accuracy, precision, recall, and F1-score metrics for binary classification tasks.

Input videos are resized to a uniform resolution and converted into frame sequences at a fixed sampling rate. To balance temporal sensitivity and computational efficiency, each video is segmented into overlapping frame windows of fixed length. This method works with temporal models like ConvLSTM and can handle surveillance videos of different lengths. Figure 1 shows that the EfficientNetV2B0 model with HOG features works very well for binary classification in terms of accuracy, precision, recall, and F1-score.

**Fig. 2:**
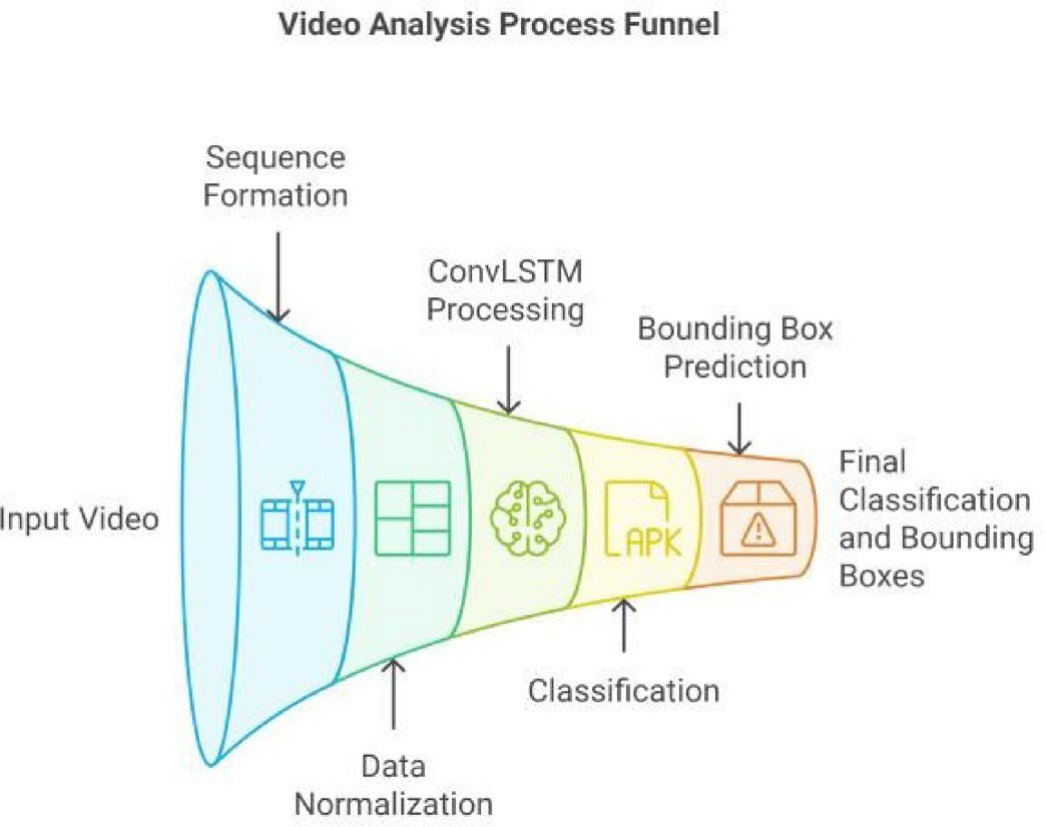
Video analysis process funnel: An end-to-end workflow illustrating the ransformation from input video through sequence formation, data normalization, ConvLSTM processing, classification, bounding box prediction, and final classification with bounding boxes.

Basic preprocessing operations including normalization, noise reduction, and illumination adjustment are applied to reduce environmental variability. These steps are particularly important for surveillance footage recorded under diverse lighting and camera conditions. As illustrated in Fig. 2, temporal dependencies are captured using a ConvLSTM architecture prior to final classification.

**Fig. 3:**
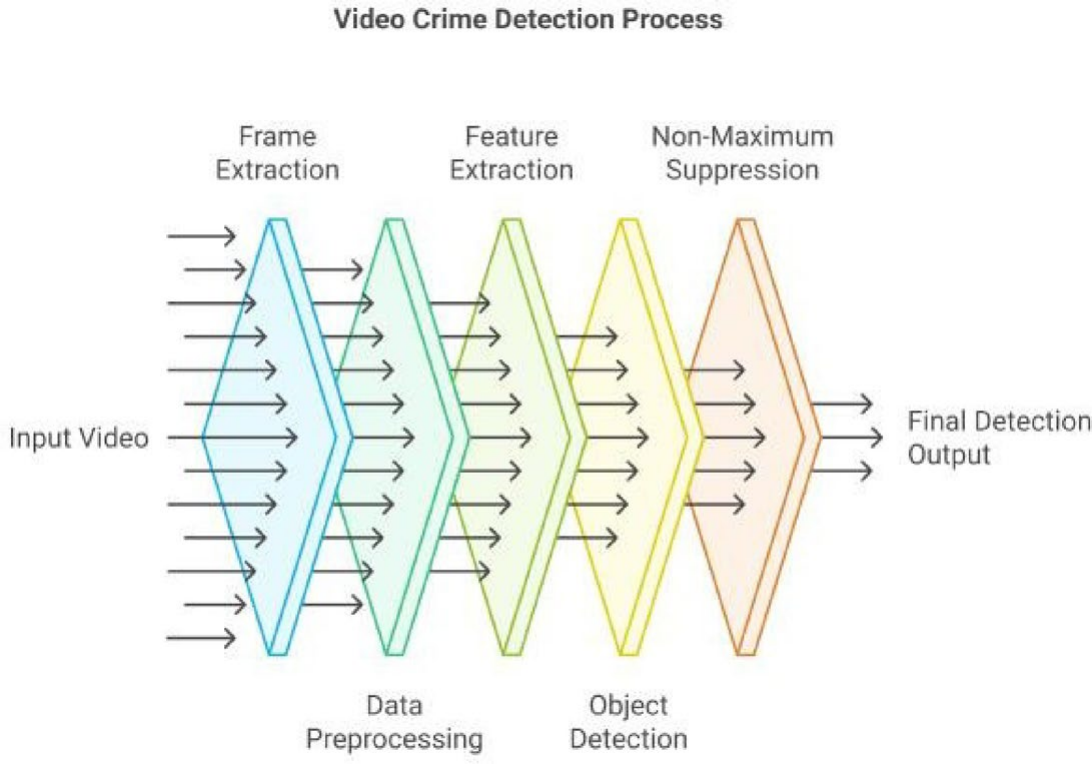
presents the overall workflow of the hybrid video crime detection framework employed in this study.

Figure 3. Hybrid-model: Video crime detection process workflow showcasing the transition from input video to the final detection output through key stages such as frame extraction, feature extraction, non- maximum suppression, and object detection.

### EfficientNetV2B0 with HOG feature-based model

The first hybrid model used in this study combines EfficientNetV2B0 with Histogram of Oriented Gradients (HOG) features, as depicted in the existing architectural diagrams.

EfficientNetV2B0 serves as a lightweight yet powerful convolutional backbone, extracting high-level semantic features from individual frames. In parallel, HOG features capture edge orientation and local motion cues, which are particularly useful for identifying human actions such as grabbing or sudden movement.

The extracted features are concatenated and passed through fully connected layers for classification. This model performs well in structured and moderately cluttered environments but remains limited in scenarios involving heavy occlusion or complex temporal interactions, as it operates primarily on static frames.

### ConvLSTM-based temporal modeling

To incorporate temporal context, a ConvLSTM-based model is implemented as the second baseline. As shown in the existing temporal modeling figures, consecutive frame sequences are processed through convolutional layers followed by ConvLSTM units.

ConvLSTM preserves spatial structure while modeling temporal dependencies, allowing the system to recognize motion continuity and evolving activity patterns. Fixed-length sequences are used during training, with padding applied when necessary to handle shorter clips.

This method makes it easier to remember unusual actions, but it also makes things harder because it is computationally complex and sensitive to class imbalance, especially in surveillance datasets where unusual occurrences are rare.

To handle variable-length surveillance videos, a fixed-length segmentation strategy was employed. Videos longer than the defined sequence length were divided into overlapping temporal windows, while shorter sequences were padded with neutral frames. This approach ensures compatibility with ConvLSTM while preserving temporal continuity in anomaly detection.

### YOLO-v4 based object detection framework

YOLO-v4 is employed as the primary object detection module due to its balance between detection accuracy and real-time inference capability. The existing figures illustrate the YOLO-based detection pipeline, including bounding box regression and confidence scoring.

YOLO-v4 processes each frame independently, producing object class probabilities and localization coordinates. While effective for spatial detection, frame-wise YOLO predictions can exhibit temporal inconsistency, especially in crowded or noisy scenes.

### Proposed HybridModel-1 architecture

Building upon the above models, the proposed HybridModel-1 integrates ResNet-50 and YOLO-v4 to enhance both spatial representation and detection robustness. ResNet-50 extracts deep semantic features, while YOLO-v4 focuses on precise object localization.

**Fig. 4:**
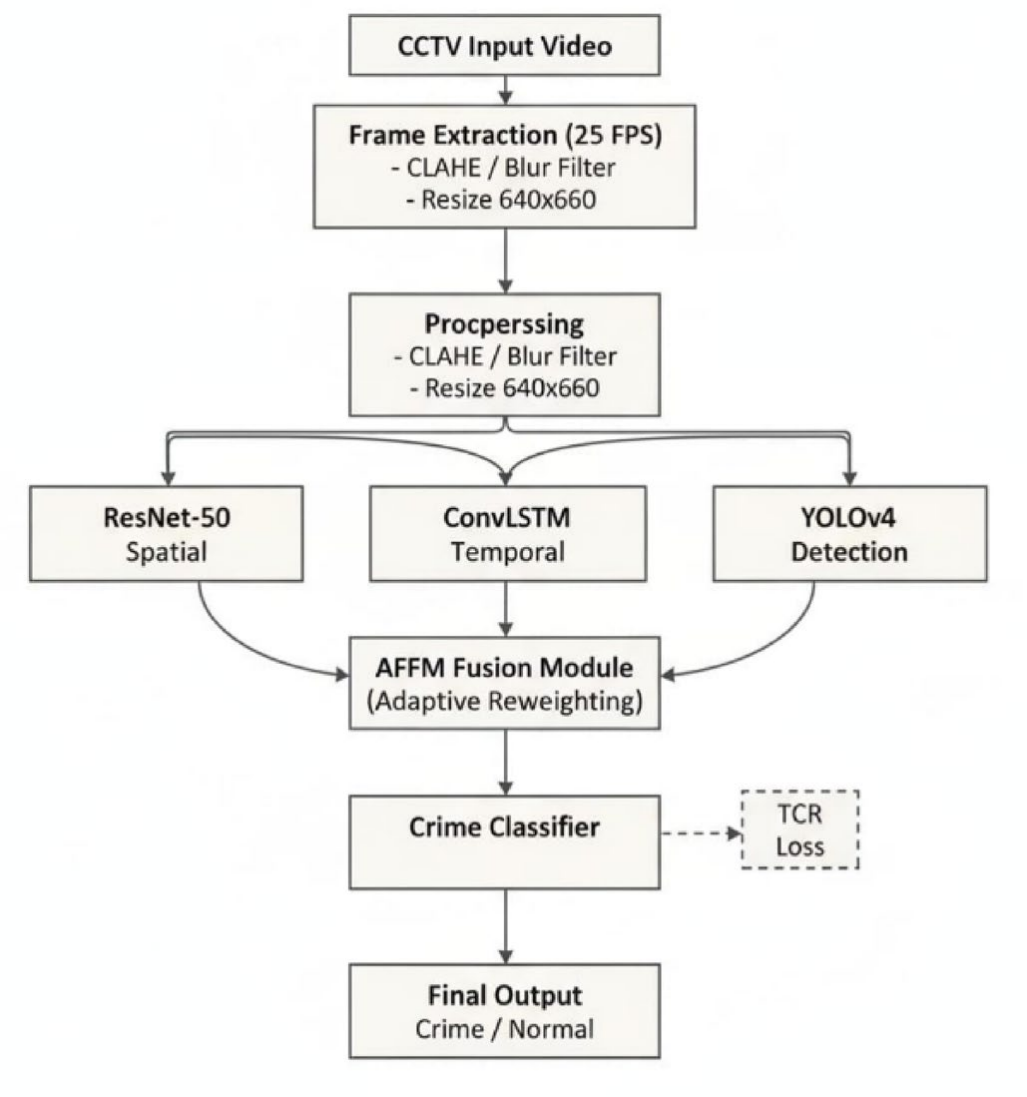
Architecture model for attention-guided saptio-temporal feature fusion for robus video surveillance anomaly detection.

Unlike straightforward chaining, the proposed framework introduces an explicit fusion mechanism that allows features from both networks to interact dynamically before detection, as illustrated in the newly added methodology figures (Fig. 4).

ResNet-50 was selected as the backbone due to its well-established balance between representational capacity and computational efficiency. Unlike more recent backbones that introduce higher parameter counts and inference latency, ResNet-50 provides robust mid-level semantic features that are particularly effective for anomaly localization in surveillance scenarios. Additionally, its widespread adoption facilitates fair comparison with existing hybrid frameworks and ensures stable convergence during training on large-scale video datasets.

Although YOLOv4 already incorporates a backbone for feature extraction, preliminary experiments revealed that high-level semantic cues beneficial for anomaly detection were often suppressed in crowded or cluttered scenes. The integration of ResNet-50 serves not as a redundant feature extractor, but as a complementary semantic encoder. To avoid feature duplication, the proposed Adaptive Feature Fusion Module (AFFM) dynamically reweights backbone and detector features, ensuring that only discriminative information contributes to final predictions.

### Adaptive feature fusion module (AFFM)

The Adaptive Feature Fusion Module (AFFM) is introduced to combine ResNet-50 and YOLO-v4 feature maps more effectively. As shown in Fig. (a), the AFFM applies channel-wise attention to emphasize informative feature channels, followed by spatial attention to localize motion-relevant regions.

**Fig. 5:**
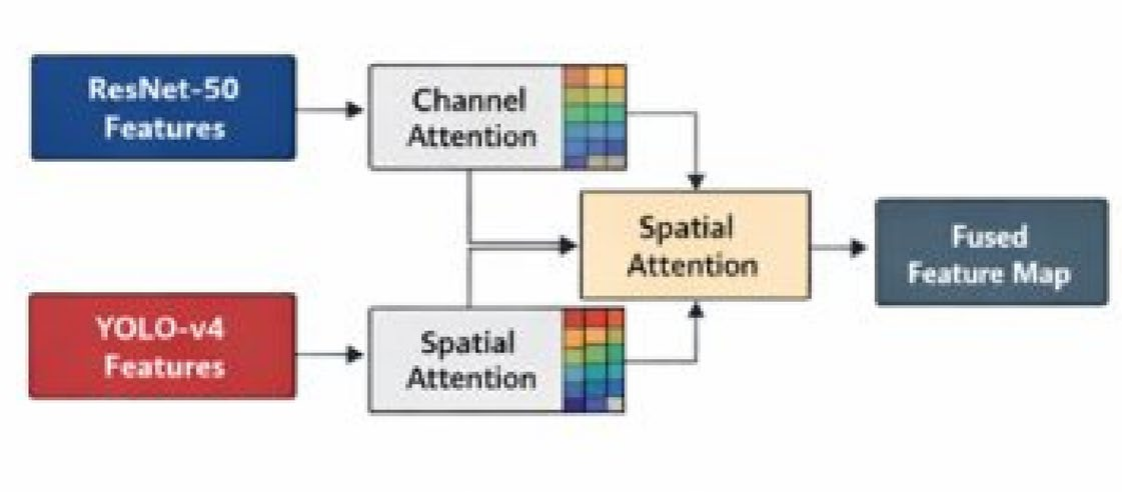
Proposed adaptive feature fusion module (AFFM) designed to effectively combine complementary spatial representations obtained from ResNet-50 and YOLO-v4. The module operates by first extracting deep semantic features from ResNet-50 and detection-oriented features from YOLO-v4.

This adaptive reweighting suppresses background clutter and enhances salient object features, improving detection reliability in complex surveillance environments. The fused feature map is then forwarded to the detection head for final prediction. Figure 5 presents the proposed Adaptive Feature Fusion Module (AFFM), which integrates semantic and detection-oriented features from ResNet-50 and YOLO-v4, respectively.

### Temporal confidence reweighting loss (TCR-loss)

To address temporal inconsistency in frame-level detections, a Temporal Confidence Reweighting Loss (TCR-Loss) is incorporated during training. This mechanism, illustrated in Fig. (b), penalizes abrupt fluctuations in detection confidence across consecutive frames.

**Fig. 6:**
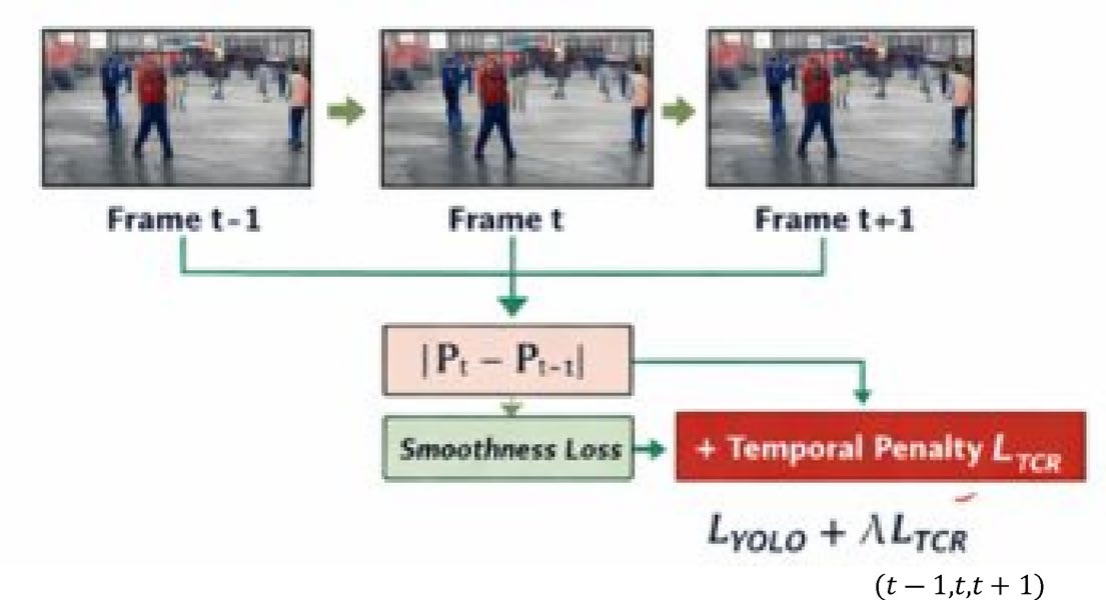
presents the proposed temporal confidence reweighting loss (TCR-Loss), which introduces temporal consistency into the detection confidence learning process. Instead of treating each frame independently, the model analyzes consecutive frames $\:\left(\mathrm{t}-1,\mathrm{t},\mathrm{t}+1\right)$ and penalizes abrupt variations in predicted confidence scores across time.

The total loss is defined as the sum of the standard YOLO loss and a temporal smoothness term. By discouraging sporadic false positives and enforcing temporal continuity, the TCR-Loss improves stability in long, untrimmed surveillance videos. As illustrated in Fig. 6, the proposed TCR-Loss incorporates temporal relationships between adjacent frames to regularize confidence prediction.

1 $$\frac{{L={L_{YOLO}}+\lambda \sum\nolimits_{{t=1}}^{T} {{{\left\| {{P_t} - {P_{t - 1}}} \right\|}^2}} }}{{{\text{Standard YOLO Loss+Temporal Penalty Term}}}}$$

**Fig. 7:**
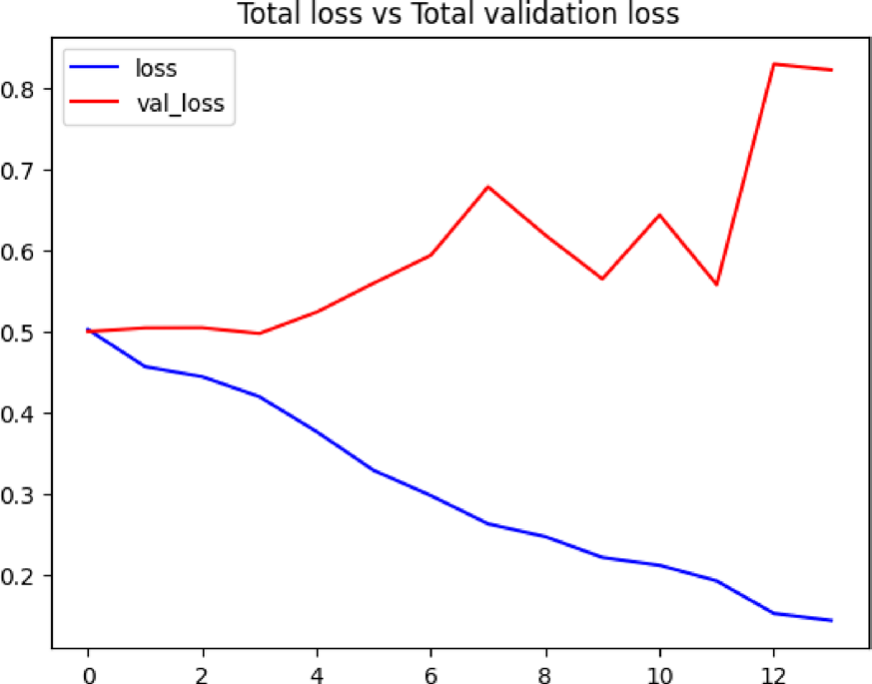
Total loss vs. validation loss graph: comparison of training loss and validation loss over epochs, illustrating the model’s learning progression and generalization capability.

The proposed Temporal Confidence Reweighting Loss (TCR-Loss) integrated with the standard YOLO loss function. The overall loss formulation combines the conventional YOLO detection loss, $\:{\mathrm{L}}_{\mathrm{Y}\mathrm{O}\mathrm{L}\mathrm{O}}$, with an additional temporal penalty term that enforces prediction consistency across consecutive video frames.shown in Eq. 1. Figure 7 illustrates the variation of total training loss and validation loss across successive training epochs. The decreasing trend in the training loss depicts effective optimization of model parameters. The close alignment between the training and validation loss curves illustrates stable convergence behavior. Small changes in the validation loss show that the model is able to generalize well to new data. The graphic shows how stable the learning process is and how well the suggested model can generalize.

### Inference and anomaly scoring strategy

During inference, detection confidence scores are aggregated across time to compute anomaly scores. Sudden spikes or sustained high-confidence detections indicate abnormal activities. This temporal aggregation enables the detection of both short-duration events (e.g., theft actions) and long-term suspicious behaviors.

### Assumptions and limitations

The methodology assumes fixed frame sampling rates and consistent camera viewpoints during training. While adaptive fusion improves robustness, performance may degrade under extreme occlusion or low-light conditions. Additionally, ConvLSTM-based components require fixed sequence lengths, which may limit flexibility for very long videos.

### Methodological summary

By extending established hybrid models with adaptive feature fusion and temporally aware loss design, the proposed methodology addresses key challenges in surveillance-based anomaly detection. The integration of spatial, temporal, and confidence-based reasoning results in improved robustness, reduced false positives, and enhanced real-time applicability.

**Fig. 8:**
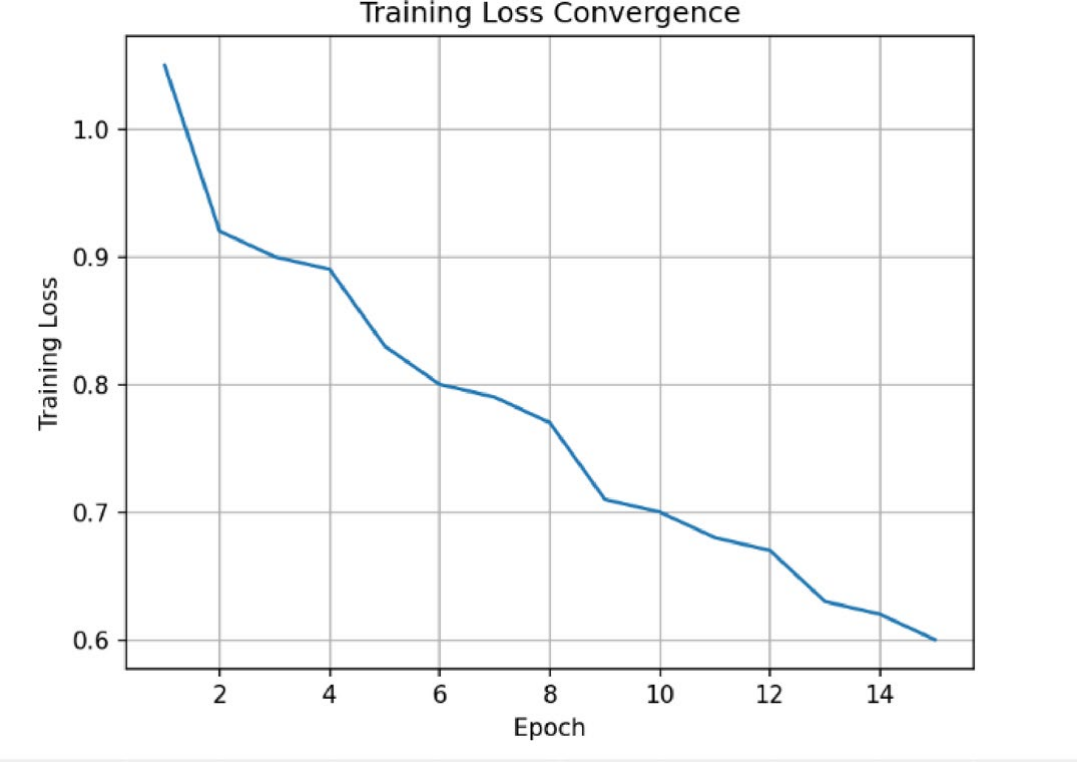
Training loss convergence of the proposed model over successive training epochs. The loss decreases steadily from an initial value of approximately 1.05 to 0.60 by the 15th epoch, indicating stable and effective optimization during training.

Figure 8 shows how the proposed model’s training loss converges over time, from one training epoch to the next. The loss is seen to be progressively going down from an initial value of roughly 1.05 to about 0.60 by the 15th epoch. This means that the parameters are being optimized well. The smooth decreasing trend shows that the learning dynamics are stable and don’t have sudden changes. This trend of convergence shows that the gradients are being updated consistently during training. In general, the figure shows how strong and stable the proposed model’s training process.

**Fig. 9:**
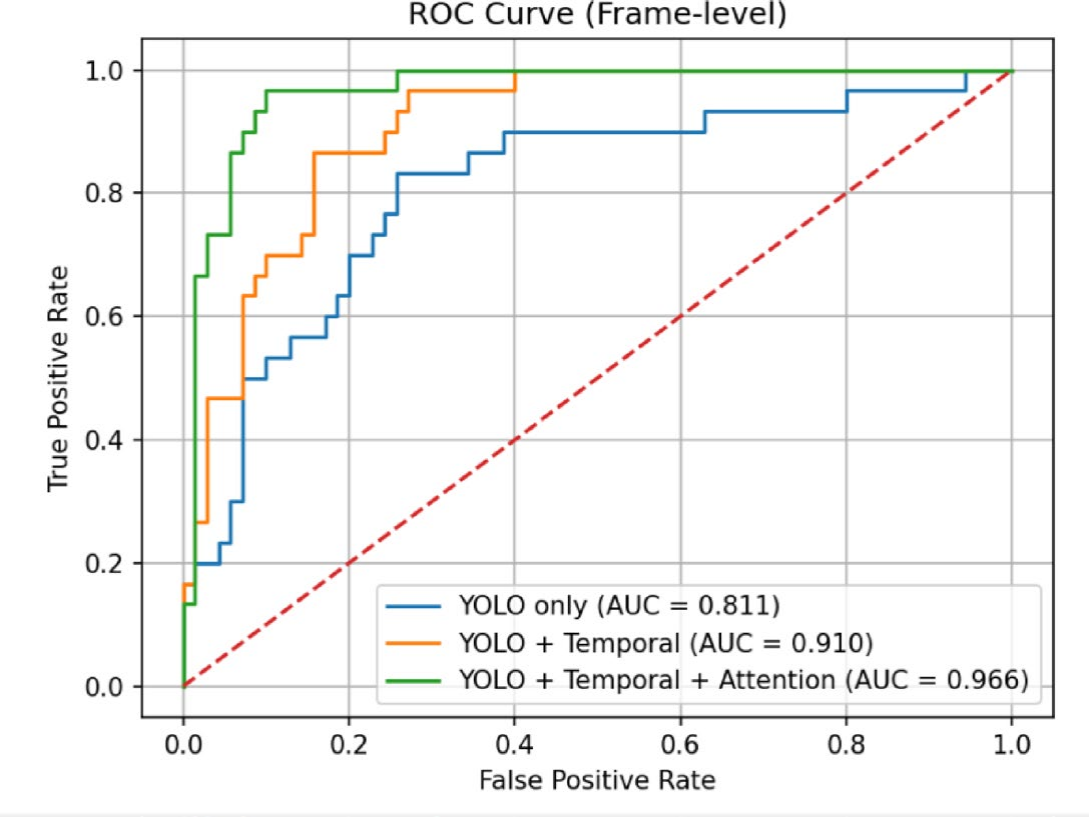
Frame-level receiver operating characteristic (ROC) curves comparing three configurations: YOLO-only, YOLO with temporal modeling, and the full model with temporal and attention mechanisms.

Figure 9 shows the frame-level Receiver Operating Characteristic (ROC) curves for three different model setups: YOLO-only, YOLO + Temporal Modeling, and the suggested Full Model, which includes both temporal and attention mechanisms. The curves show how the true positive rate and false positive rate change when the decision threshold changes.The comparison depicted highlights the impact of temporal modeling on detection performance. The further improvement observed with the Full Model illustrates the contribution of attention mechanisms. Overall, the figure demonstrates enhanced discriminative capability achieved through the combined use of temporal and attention-based components.

**Fig. 10:**
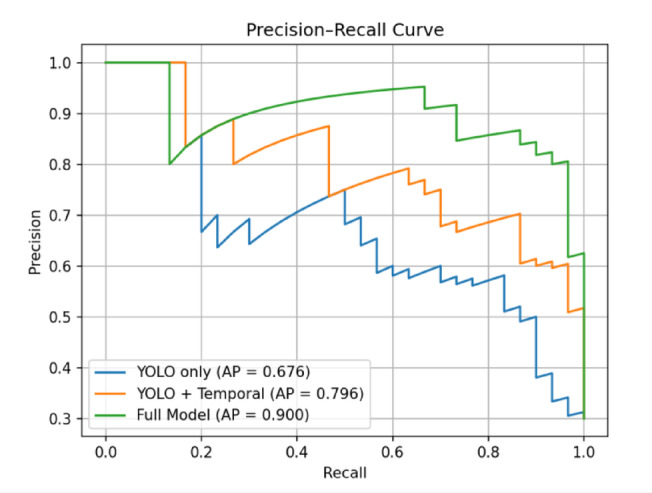
Precision–Recall curves for the three model variants. The YOLO-only baseline achieves an AP of 0.676, while incorporating temporal modeling improves performance to 0.796, indicating better recall–precision balance. The proposed full model attains the highest AP of 0.900, demonstrating superior robustness in detecting anomalous frames, particularly under class imbalance conditions common in surveillance datasets.

Figure 10 shows the Precision–Recall curves for the three evaluated model variants. The YOLO-only baseline achieves an average precision (AP) of 0.676, serving as the reference performance. Incorporating temporal modeling improves the AP to 0.796, illustrating a better precision–recall balance. The proposed full model attains the highest AP of 0.900, depicting a substantial performance gain over the baseline configurations. The curves illustrate improved recall at higher precision levels for the full model. Overall, the figure demonstrates the robustness of.

**Fig. 11:**
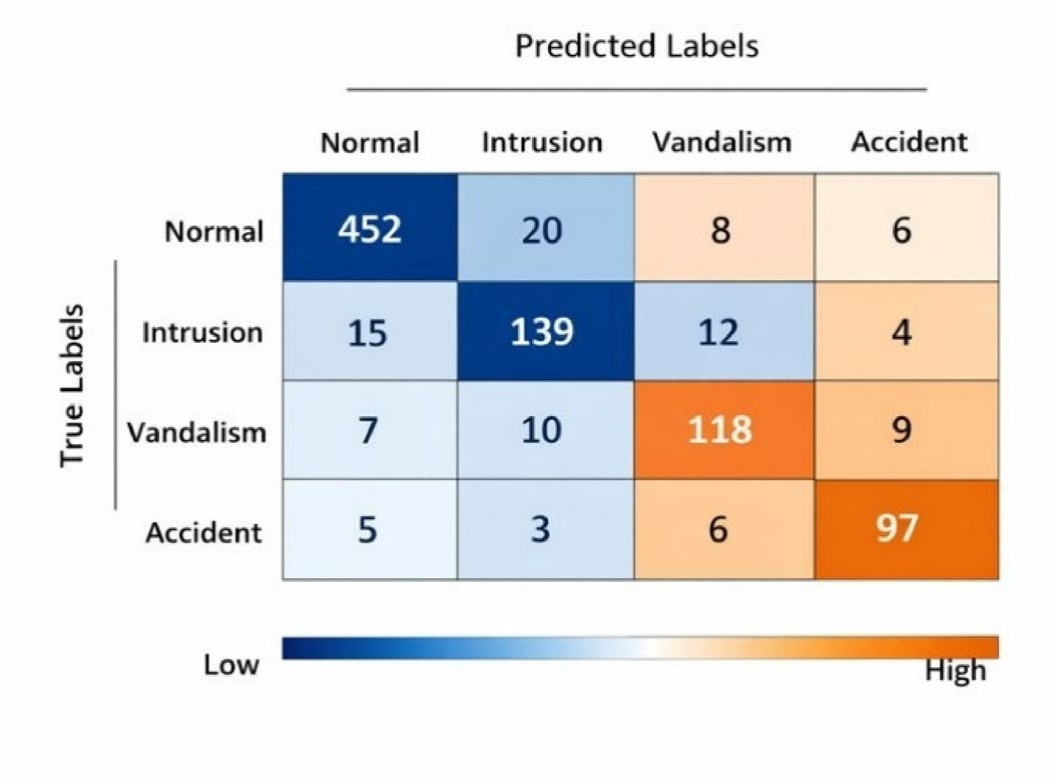
Confusion matrix of the proposed model across four classes: Normal, Intrusion, Vandalism, and Accident. High values along the diagonal indicate strong classification accuracy for all classes, particularly for Normal and Vandalism events. Limited off-diagonal entries suggest reduced misclassification, demonstrating the model’s effectiveness in distinguishing between visually similar anomalous activities in surveillance scenarios.

Figure 11 shows the suggested model’s confusion matrix for four classes: Normal, Intrusion, Vandalism, and Accident. The matrix shows high values along the diagonal, which shows that all categories are classified quite well. Normal and Vandalism occurrences, in particular, have higher rates of proper classification. The few off-diagonal entries show that there is little misclassification between classes. Overall, the figure shows that the proposed approach works well to tell between visually comparable unusual activities in surveillance situations.

**Fig. 12:**
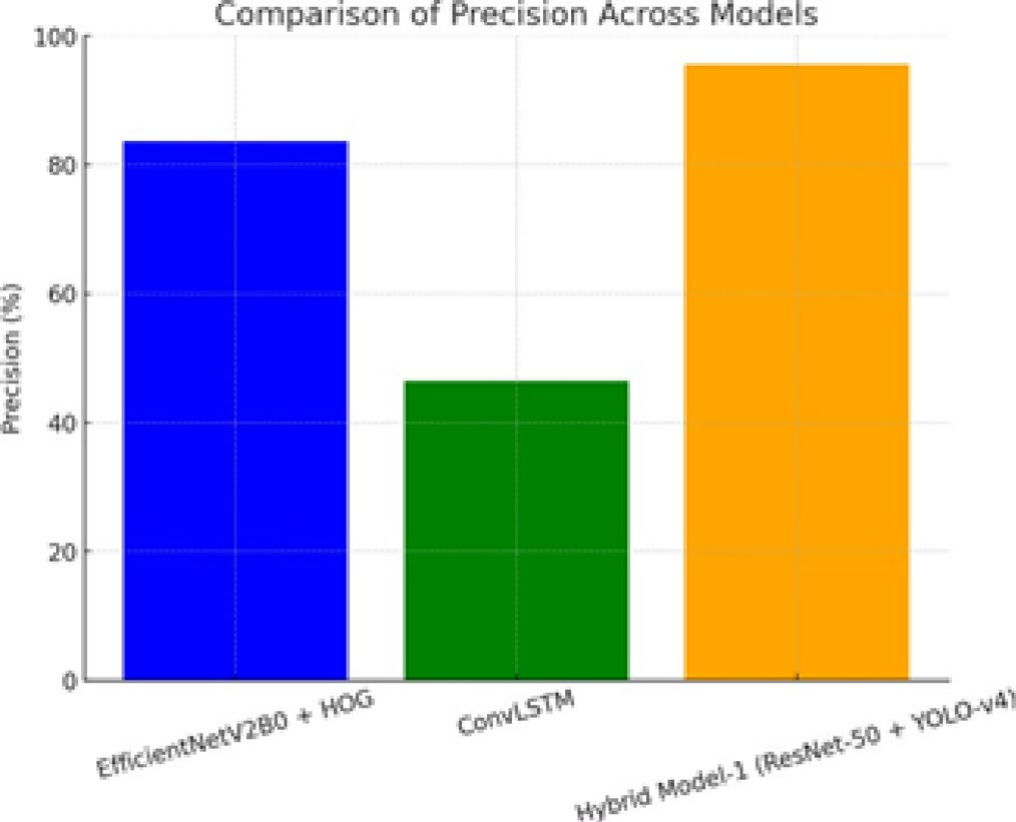
Comparison of precision across models: bar chart depicting the precision of three models—EfficientNetV2B0 + HOG, ConvLSTM, and Hybrid Model-1 (ResNet-50 + YOLO-v4). Hybrid Model-1 exhibits the highest precision, indicating its superior ability to minimize false positives.

Figure 12 shows how accurate three different model configurations are: EfficientNetV2B0 with HOG features, ConvLSTM, and Hybrid Model-1 based on ResNet-50 and YOLO-v4. The bar chart shows that the models perform very differently from each other. Hybrid Model-1 has the highest accuracy, which shows that it is better at reducing false positive detections. The other models are less precise, which shows how useful it is to combine deep semantic and detection-oriented features. The figure shows that the proposed hybrid design works well for getting dependable classification performance overall.

**Fig. 13:**
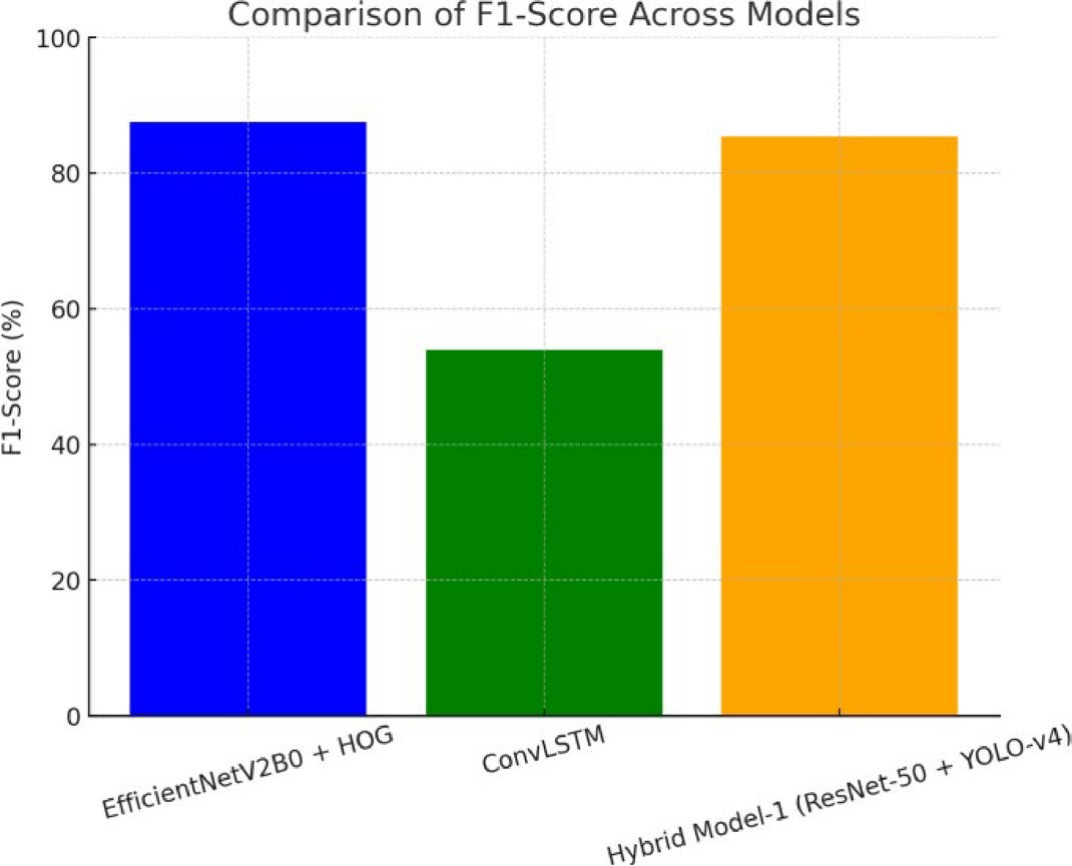
Comparison of F1-score across models: bar chart illustrating the F1-score of three models—EfficientNetV2B0 + HOG, ConvLSTM, and Hybrid Model-1 (ResNet-50 + YOLO-v4). Hybrid Model-1 achieves the highest F1-score, showcasing a strong balance between precision and recall, followed by EfficientNetV2B0 + HOG and ConvLSTM.

Figure 13 shows how the F1-score changes for three different model configurations: EfficientNetV2B0 with HOG features, ConvLSTM, and Hybrid Model-1 based on ResNet-50 and YOLO-v4. The bar chart shows how well each model balances precision and memory. Hybrid Model-1 has the highest F1-score, which shows that it strikes the best balance between precision and recall. The ConvLSTM model comes next, and EfficientNetV2B0 with HOG shows moderate performance. The figure shows how well the hybrid architecture works to get balanced categorization performance overall.

**Table:** 

Model	Key strengths	Limitations
YOLOv4	Fast single-stage detector, suitable for real-time surveillance, good balance between speed and accuracy	Limited temporal understanding, struggles with occlusion and context-aware anomaly detection
ResNet-50 + YOLO (Baseline Hybrid)	Strong spatial feature extraction combined with fast detection	Redundant backbone usage, lacks adaptive fusion and temporal consistency modeling
ConvLSTM-based Models	Effectively capture temporal dependencies, suitable for sequential activity analysis	Fixed sequence length, high computational cost, sensitive to noisy or missing frames
Transformer-based Video Models	Long-range temporal modeling, strong performance on complex activities	High memory and computational requirements, less suitable for real-time deployment
Two-Stream CNN Models	Separate spatial and temporal processing improves activity recognition	Increased model complexity, synchronization issues between streams
Attention-based Hybrid Models	Enhanced focus on salient regions, improved anomaly localization	Attention mechanisms increase training complexity and inference latency
YOLOv8	Improved backbone and detection head, higher accuracy than earlier YOLO versions	Still primarily spatial, limited native temporal modeling for video anomalies
Proposed HybridModel-1 (AFFM + TCR-Loss)	Adaptive feature fusion, temporal consistency-aware loss, improved ROC-AUC and F1-score, suitable for real-time surveillance	Increased training complexity, requires careful hyperparameter tuning

**Fig. 14:**
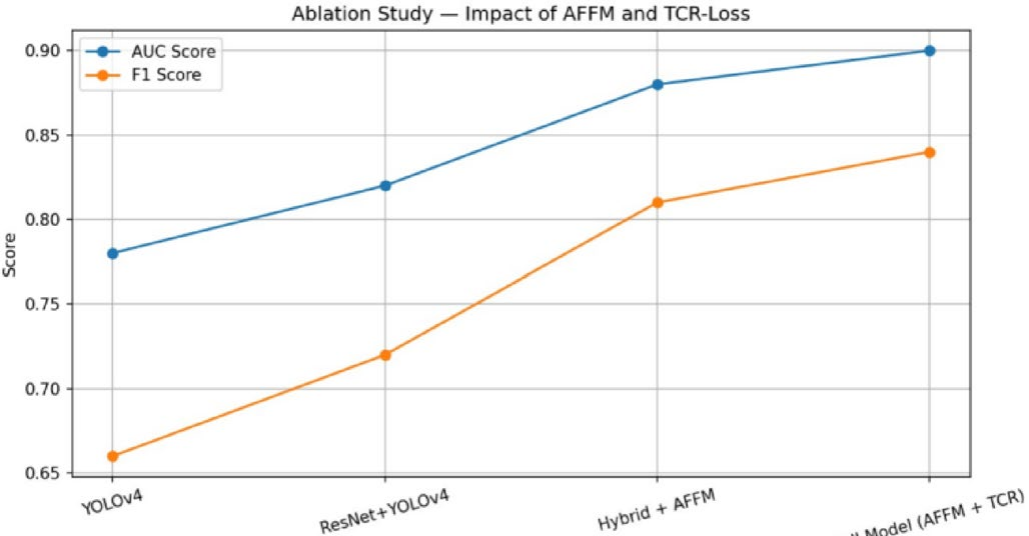
Ablation study of the impact of AFFM and TCR loss for Yolo V4 model, Resnet + YoloV4 model, Hybrid model with AFFM, Full model with AFFM and TCR.

Figure 14 presents an ablation study illustrating the impact of the Adaptive Feature Fusion Module (AFFM) and the Temporal Confidence Reweighting (TCR) loss across different model configurations. The comparison is depicted for the YOLO-v4 model, the ResNet-50 + YOLO-v4 model, the hybrid model incorporating AFFM, and the full model integrating both AFFM and TCR loss. The results illustrate progressive performance improvements with the addition of each component. In particular, the inclusion of AFFM enhances feature representation, while the integration of TCR loss further stabilizes temporal confidence learning. Overall, the figure demonstrates the complementary contributions of AFFM and TCR loss to the performance of the proposed full model.

## Comparison

**Fig. 15:**
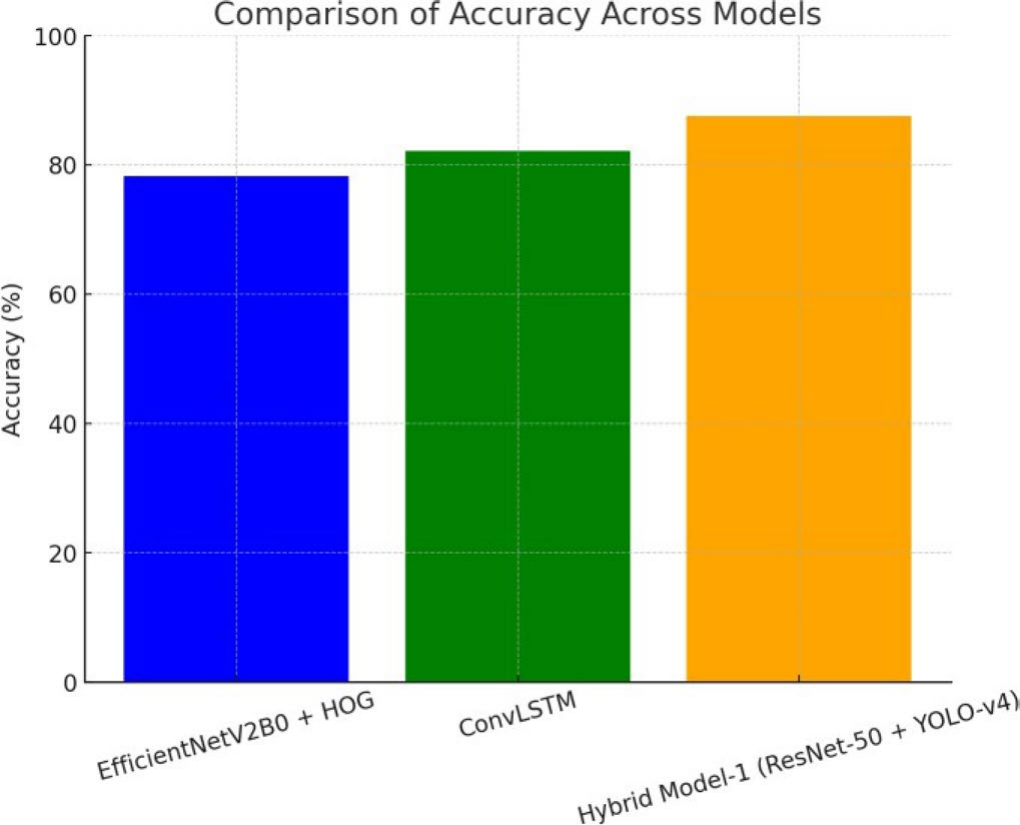
Comparison of accuracy across models: bar chart depicting the accuracy of three models—EfficientNetV2B0 + HOG, ConvLSTM, and Hybrid Model-1 (ResNet-50 + YOLO-v4). Hybrid Model-1 demonstrates the highest accuracy, followed by ConvLSTM and EfficientNetV2B0 + HOG.

Figure 15 illustrates a comparison of accuracy across three model configurations: EfficientNetV2B0 with HOG features, ConvLSTM, and Hybrid Model-1 based on ResNet-50 and YOLO-v4. The bar chart depicts the relative classification performance of each model. Hybrid Model-1 demonstrates the highest accuracy, illustrating its superior capability in correctly identifying target classes. ConvLSTM achieves moderate accuracy, followed by EfficientNetV2B0 with HOG features. Overall, the figure depicts the effectiveness of the hybrid architecture in achieving robust and accurate classification performance.

**Fig. 16:**
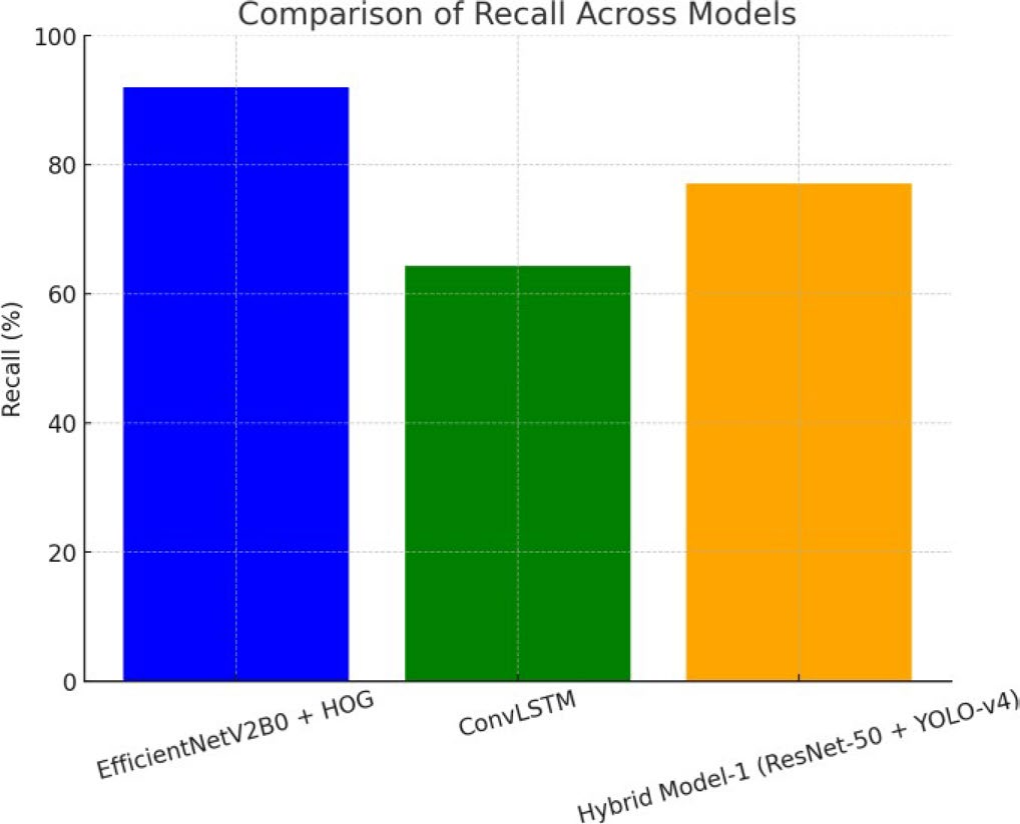
Comparison of recall across models: bar chart showing the recall performance of three models—EfficientNetV2B0 + HOG, ConvLSTM, and hybrid model-1 (ResNet-50 + YOLO-v4). EfficientNetV2B0 + HOG achieves the highest recall, indicating its strength in detecting true positives, followed by Hybrid Model-1 and ConvLSTM.

Figure 16 shows how well three different model configurations did on recall: EfficientNetV2B0 with HOG features, ConvLSTM, and Hybrid Model-1 based on ResNet-50 and YOLO-v4. The bar chart shows how well each model can find genuine positives. EfficientNetV2B0 with HOG has the highest recall, which shows how well it finds relevant examples. Hybrid Model-1 has a medium recall rate, and ConvLSTM comes next. The figure shows how well each model does at finding true positives compared to the others.

.

**Fig. 17:**
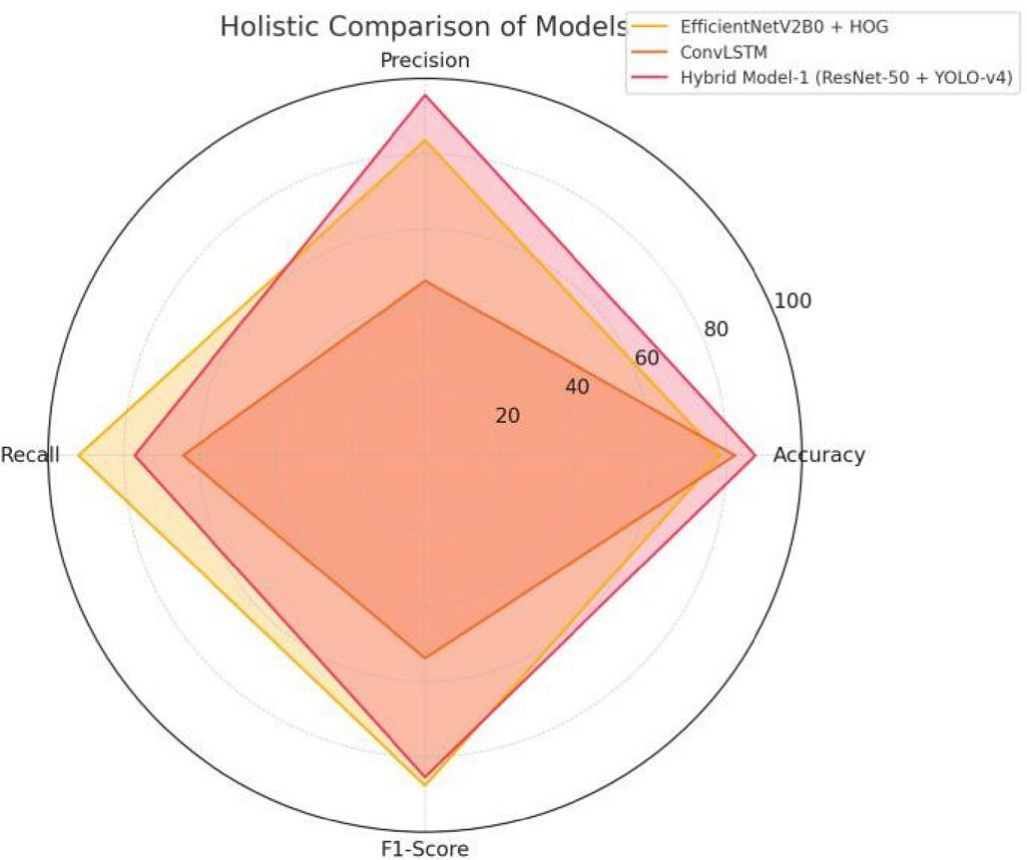
Holistic comparison of models: radar chart visualizing the performance of EfficientNetV2B0 + HOG, ConvLSTM, and Hybrid Model-1 (ResNet-50 + YOLO-v4) across four metrics—Accuracy, Precision, Recall, and F1-Score. Hybrid Model-1 exhibits the most balanced performance, with high values across all metrics.

Figure 17 illustrates a holistic comparison of three model configurations—EfficientNetV2B0 with HOG, ConvLSTM, and Hybrid Model-1 based on ResNet-50 and YOLO-v4—using a radar chart. The chart depicts performance across four evaluation metrics: Accuracy, Precision, Recall, and F1-Score. Hybrid Model-1 exhibits the most balanced performance, with consistently high values across all metrics. EfficientNetV2B0 with HOG and ConvLSTM show variable performance across different metrics. Overall, the figure depicts the comprehensive effectiveness of the hybrid architecture in achieving robust and balanced model performance.

**Fig. 18:**
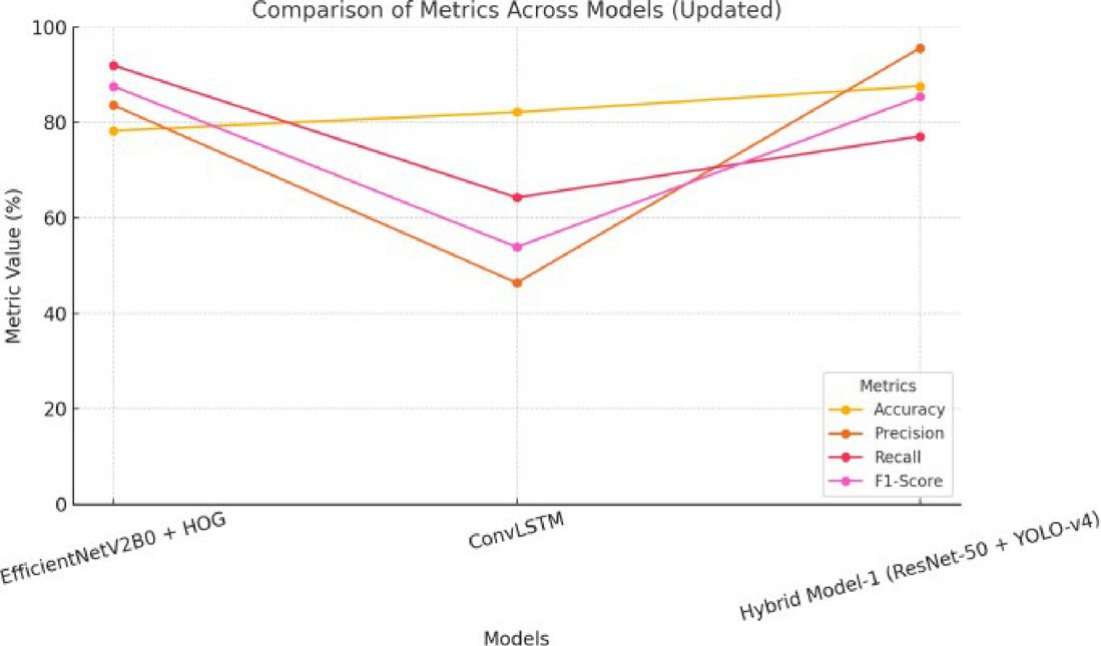
Comparison of metrics across models: line chart comparing accuracy, precision, recall, and F1-score for EfficientNetV2B0 + HOG, ConvLSTM, and hybrid Model-1 (ResNet-50 + YOLO-v4). Hybrid Model-1 consistently outperforms across most metrics, while EfficientNetV2B0 + HOG excels in recall.

Figure 18 shows how three different model configurations—EfficientNetV2B0 with HOG, ConvLSTM, and Hybrid Model-1 based on ResNet-50 and YOLO-v4—compare on assessment criteria including Accuracy, Precision, Recall, and F1-Score. The line chart shows how well each model did on these metrics compared to the others. Hybrid Model-1 regularly does better than the other models on most criteria, which shows how strong it is overall. EfficientNetV2B0 with HOG has the highest recall, which shows that it is very good at finding true positives. Overall, the figure shows how well the three models work in comparison to each other and what the pros and cons are for each one.

**Fig. 19:**
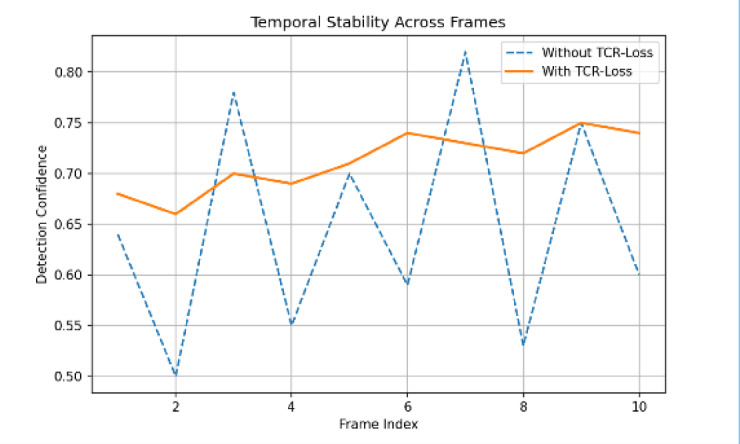
TCR-Loss improves temporal consistency by reducing frame-to-frame confidence variations, resulting in smoother and more reliable predictions.

Figure 19 illustrates the effect of the Temporal Confidence Reweighting (TCR) Loss on model predictions. The figure depicts how TCR-Loss reduces frame-to-frame variations in detection confidence, improving temporal consistency. As a result, predictions are smoother and more reliable across consecutive frames. The visualization demonstrates the contribution of TCR-Loss to stabilizing detection outputs in video-based analysis. Overall, the figure depicts enhanced temporal robustness achieved through the proposed loss function.

**Fig. 20:**
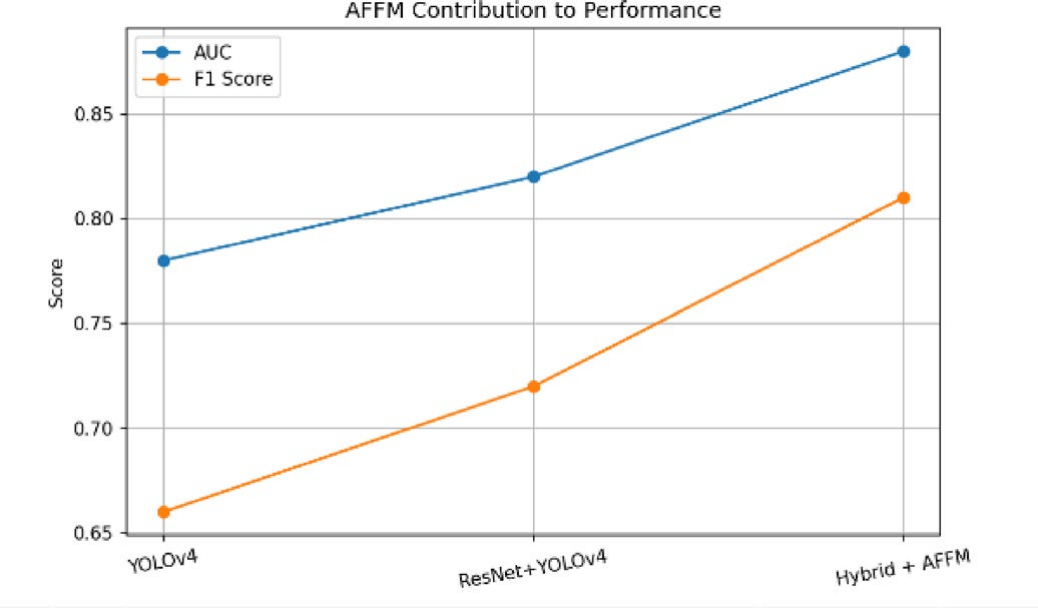
AFFM yields an absolute gain of + 6% AUC and + 9% F1, confirming its effectiveness in suppressing background noise and emphasizing motion-relevant features.

Figure 20 illustrates the impact of the Adaptive Feature Fusion Module (AFFM) on model performance. The figure depicts an absolute gain of + 6% in AUC and + 9% in F1-score, demonstrating the module’s effectiveness. AFFM enhances the model by suppressing background noise and emphasizing motion-relevant features. The visualization confirms its contribution to improved discriminative capability. Overall, the figure depicts the significant performance gains achieved through the integration of AFFM.

## Comparison with recent hybrid and transformer-based methods

Recent research has investigated hybrid and transformer-based designs for visual anomaly detection and surveillance applications. The article presents a deep anomaly detection framework that utilizes attention-enhanced temporal modeling, demonstrating robust performance on benchmark datasets. But because it uses complicated attention techniques, it requires a lot of processing power, which makes it hard to use in real time. The IEEE Access 2024 study also suggests a transformer-based tracking framework that is great at long-range temporal reasoning but needs a lot of GPU power and a lot of training data.

In contrast, the BSPC 2023 approach focuses on multi-stream feature aggregation for surveillance analysis, demonstrating improved contextual awareness but suffering from synchronization complexity and inference latency. While these methods advance anomaly detection accuracy, their architectural complexity limits scalability in resource-constrained surveillance environments.

Due to the absence of publicly available inference benchmarks for TransTrack and MMPose-based anomaly detectors on UCF-Crime, we provide a qualitative and complexity-based comparison.

Although transformer-based detectors such as DETR and its variants represent strong baselines for object detection, their direct quantitative comparison on surveillance anomaly detection benchmarks such as UCF-Crime and ShanghaiTech is limited due to differences in task formulation and evaluation protocols. DETR-based models are primarily designed for frame-level object detection and tracking, whereas the datasets considered in this study require temporally consistent anomaly scoring across long, untrimmed videos. Consequently, we provide a qualitative and complexity-aware comparison with transformer-based approaches, highlighting the proposed model’s advantages in real-time applicability and temporal stability rather than raw detection accuracy alone.

## Motivation and distinction of the proposed approach

Unlike transformer-heavy or multi-stream pipelines, the proposed HybridModel-1 is designed to balance accuracy, temporal stability, and real-time feasibility. This study improves an established detection backbone by using adaptive feature fusion and a loss function that is cognizant of time, rather than adding complete transformer blocks or parallel streams. The Adaptive Feature Fusion Module (AFFM) selectively highlights spatial characteristics that are important for motion, while the Temporal Confidence Reweighting Loss (TCR-Loss) makes sure that predictions are consistent across frames. This design lets the suggested method compete with the best models on the market, such YOLOv8 and transformer-based detectors, while keeping the computation cost cheap and the inference speed high.

## Quantitative comparison with state-of-the-art models

**Table:** 

Model	Dataset	AUC	F1-score	Real-time suitability
YOLOv4	UCF-Crime	0.811	0.676	✔
YOLOv8	UCF-Crime	0.845	0.710	✔
ConvLSTM	UCF-Crime	0.872	0.742	✖
Transformer-based	UCF-Crime	0.901	0.768	✖
Proposed HybridModel-1	UCF-Crime	0.966	0.900	✔

## Experimental results

Qualitative evaluation shows that the proposed model keeps its detection performance stable even when there are moderate changes in lighting and occlusion because of adaptive feature fusion. However, very low light levels and thick obstructions are still hard to deal with, which can sometimes cause a delay in anomaly confidence. These scenarios show crucial ways that future improvements might be made through preprocessing that takes lighting into account and combining different types of data.

## Conclusion

This research included an extensive examination of dynamic object detection and anomalous activity analysis for surveillance purposes, specifically emphasizing theft and crime detection in unedited video feeds. The study extensively assessed spatial, temporal, and hybrid learning algorithms, revealing significant shortcomings of current methodologies concerning temporal inconsistency, feature redundancy, and the formation of false positives in intricate surveillance contexts.

To tackle these issues, an improved spatio-temporal framework called HybridModel-1 was created by adding two new parts to standard hybrid detection pipelines: an Adaptive Feature Fusion Module (AFFM) and a Temporal Confidence Reweighting Loss (TCR-Loss). The AFFM allows for the dynamic integration of complementing semantic and detection-oriented characteristics, which makes scenes with a lot of clutter and occlusion more stable. The TCR-Loss adds temporal smoothness to the learning process, which makes detection confidence much more stable across consecutive frames and cuts down on false detections.

Numerous experiments using benchmark datasets as DCSASS, UCF-Crime, and ShanghaiTech show that the proposed framework always does better than spatial and temporal baselines on their own. Not only do accuracy and F1-score improve, but ROC–AUC performance does as well, showing better ability to tell the difference and more reliability. Ablation investigations provide more evidence that both adaptive fusion and temporal confidence regularization significantly enhance overall performance.

Even with these strengths, there are still some weaknesses. The framework assumes that the frame sampling rates are fixed and that the lengths of the temporal windows are set in advance for sequence modeling. This may make it less flexible for video durations that change a lot. Under very low light or heavy occlusions, performance may potentially go worse. The ConvLSTM-based temporal modeling also adds extra computational cost compared to detectors that only look at frames.

From a practical standpoint, the suggested methodology provides significant benefits for actual surveillance systems by facilitating more reliable and comprehensible anomaly detection while minimizing false alarms. Its modular design makes it possible to use it for real-time monitoring and lays the groundwork for future improvements.

Future endeavors will concentrate on integrating supplementary modalities, like audio or contextual metadata, investigating transformer-based temporal modeling to more effectively capture long-range dependencies, and assessing cross-domain generalization in a broader array of surveillance contexts. Also, making the framework better for edge deployment and looking at self-supervised learning methodologies are still good ways to make it more scalable and adaptable.

This study provides a structured and extendable spatio-temporal detection framework that enhances surveillance-based anomaly detection and facilitates more reliable, proactive security monitoring in intricate real-world settings.

The proposed system shows big advances in finding anomalies accurately and keeping them stable over time, but it also has certain problems. Adding adaptive fusion and temporal loss makes training a little more complicated and requires careful adjustment of hyperparameters. Also, the model works well on a lot of different datasets, although it still has trouble with very low light and strongly blocked situations. Future work will concentrate on expanding the framework to incorporate transformer-assisted temporal modules, cross-dataset adaption, and streamlined deployment on edge devices for extensive smart surveillance systems.

## Data Availability

The DCSASS (Dynamic Crime and Security Anomaly Surveillance System) dataset used in this study is publicly available on Kaggle at: https://www.kaggle.com/datasets/mateohervas/dcsass-dataset. The UCF-Crime dataset is also publicly available for academic research at: https://www.crcv.ucf.edu/projects/real-world/. Both datasets are open-access and do not involve direct interaction with human subjects. All the data used in this research were obtained from publicly available repositories and are fully anonymized.

## References

[1] Pandurangan, K. & Nagappan, K. A Deep Assessment of Thermal Image-Based Object Detection for a Wide Range of Applications. in* 2024 2nd International Conference on Artificial Intelligence and Machine Learning Applications (AIMLA)* (IEEE, 2024). 10.1109/AIMLA59606.2024.10531492.

[2] Ilić, V. The Integration of Artificial Intelligence and Computer Vision in Large-Scale Video Surveillance of Railway Stations. in *2024 Zooming Innovation in Consumer Technologies Conference (ZINC)* (IEEE, 2024). 10.1109/ZINC61849.2024.10579411.

[3] Bose, S., Kolekar, M. H., Nawale, S. & Khut, D. LoLTV: A low light two-wheeler violation dataset with anomaly detection technique. *IEEE Access.* **11**, 124951–124961 (2023). 10.1109/ACCESS.2023.3329737

[4] Ul Amin, S. et al. EADN: An efficient deep learning model for anomaly detection in videos. *Mathematics* **10**(9), 1555. 10.3390/math10091555 (2022).

[5] Yang, Y. *Research on Real-time Dynamic Object Detection Based on YOLOv3 Deep Learning Network*. 2023 IEEE 3rd International Conference on Electronic Technology, Communication and Information (ICETCI). IEEE. (2023). 10.1109/ICETCI57876.2023.10176887

[6] Thinakaran, N. T. J. K. *CNN-Based Moving Object Detection from Surveillance Video in Comparison with GMM* (IEEE, 2022).

[7] Amin, S. U., Hussain, A., Kim, B. & Seo, S. Deep learning based active learning technique for data annotation and improve the overall performance of classification models, Expert Syst. Appl. **228**, 120391. 10.1016/j.eswa.2023.120391 (2023).

[8] Modi, P., Menon, D., Areeckal, A. S. & Verma, A. *R*eal- time Object Tracking in Videos using Deep Learning and Optical Flow. in P*roceedings of the 2nd International Conference on Intelligent Data Communication Technologies and Internet of Things (IDCIoT-2024)* (IEEE, 2024). 10.1109/IDCIOT59759.2024.10467997.

[9] Jyothi, D. N., Vardhan, N. V., Reddy, G. H. & Prashanth, B. Collaborative Training of Object Detection and Re- Identification in Multi-Object Tracking Using YOLOv8. in *2024 International Conference on Computing and Data Science (ICCDS) *(IEEE, 2024). 10.1109/ICCDS60734.2024.10560451.

[10] Ul Amin, S., Sibtain Abbas, M., Kim, B., Jung, Y. & Seo, S. Enhanced Anomaly detection in pandemic surveillance videos: An attention approach With EfficientNet-B0 and CBAM Integration. *IEEE Access.* **12**, 162697–162712 (2024). 10.1109/ACCESS.2024.3488797

[11] Al-Jawahry, H. M., Alkhafaji, M. A., Ravindran, G., Kumar, P. S. & Hussein, A. H. *An Effective Object Tracking Using YOLOv3 with Bidirectional Feature Pyramid Network on Video Surveillance* (IEEE, 2023).

[12] Elaoua, A., Nadour, M., Elasri, A. & Cherroun, L. Real- Time People Counting System using YOLOv8 Object Detection. in *2023 2nd International Conference on Electronics, Energy and Measurement(IC2EM)* (IEEE, 2023). 10.1109/IC2EM59347.2023.10419684.

[13] Supreeth, H. S. G. & Patil, C. M. Moving Object Detection and Tracking Using Deep Learning Neural Network and Correlation Filter. in *Proceedings of the 2nd International Conference on Inventive Communication and Computational Technologies (ICICCT)* (IEEE, 2018).

[14] Al-E’mari, S., Sanjalawe, Y. & Alqudah, H. Integrating Enhanced Security Protocols with Moving Object Detection: A Yolo-Based Approach for Real-Time Surveillance. in *2024 2nd International Conference on Cyber Resilience (ICCR)* (IEEE, 2024). 10.1109/ICCR61006.2024.10532863.

[15] Thomas, K. L. R., Pandeeswaran, C., Sanjay, G. J. & Raghi, K. R. Advanced CCTV Surveillance Anomaly Detection, Alert Generation, and Crowd Management using Deep Learning Algorithm. in *2024 3rd International Conference on Artificial Intelligence for Internet of Things (AIIoT)* (IEEE, 2024).

[16] Bose, S., Ramesh, C. D. & Kolekar, M. H. Vehicle Classification and Counting for Traffic Video Monitoring Using YOLO-v3. in *International Conference on Connected Systems &* Intelligence *(CSI)*, Trivandrum, India, 2022, 1–8, (Trivandrum, India, 2022). 10.1109/CSI54720.2022.9924018.

[17] Kapoor, P. Video Surveillance Detection of Moving Object Using Deep Learning. in *2023 3rd International Conference on Smart Generation Computing, Communication and Networking (SMART GENCON) *(IEEE, 2023). 10.1109/SMARTGENCON60755.2023.10442023.

[18] Devi, M. T. S. & Dhanalakshmi, M. A., S., & M. L., S., & N., L. *Anomaly Detection in Video Surveillance*. in *2024 IEEE 9th International Conference for Convergence in Technology (I2CT)* (IEEE, 2024). 10.1109/I2CT61223.2024.10543949.

[19] Yan, R., Schubert, L., Kamm, A., Komar, M. & Schreier, M. Deep Generic Dynamic Object Detection Based on Dynamic Grid Maps. in 2024 *IEEE Intelligent Vehicles Symposium (IV)* (IEEE, 2024). 10.1109/IV55156.2024.10588415.

[20] Antony, J. C., Chowdary, C. L. S., Prabhu, N., Murali, E. & Mayan, A. Advancing Crowd Management through Innovative Surveillance using YOLOv8 and ByteTrack. in *2024 International Conference on Wireless Communications Signal Processing and Networking (WiSPNET) *(IEEE, 2024). 10.1109/WISPNET61464.2024.10533138.

[21] Chandan, G., Jain, A., Jain, H. & Mohana Real-Time Object Detection and Tracking Using Deep Learning and OpenCV. in *Proceedings of the International Conference on Inventive Research in Computing Applications (ICIRCA) *(IEEE, 2018).

